# Microstructural Correlates of Learning in the Human Brain

**DOI:** 10.1177/10738584261435498

**Published:** 2026-03-31

**Authors:** Nico Lehmann

**Affiliations:** 1 Faculty of Humanities, Department of Sport Science, Otto von Guericke University Magdeburg, Magdeburg, Germany; 2 Collaborative Research Center 1436 ‘Neural Resources of Cognition’, Magdeburg, Germany; 3 Department of Neurology, Max Planck Institute for Human Cognitive and Brain Sciences, Leipzig, Germany

**Keywords:** neuroplasticity, brain microstructure, learning, cognition, MRI

## Abstract

Learning shapes the human brain, yet structural changes underlying this process remain difficult to characterize in vivo. Recent advances in magnetic resonance imaging (MRI)—including relaxometry, magnetization transfer, proton density, and diffusion imaging—combined with improved hardware and biophysical models, now allow highly specific assessment of subtle microstructural changes during learning. Here, we review studies documenting learning-induced changes in brain microstructure. Short training intervals elicit rapid MRI-detectable changes, including increases in restricted diffusion and local tissue volume, particularly in the hippocampus, potentially reflecting early neurite and glial adaptations. Longer training periods reveal additional changes in task-relevant gray and white matter, suggestive of adaptations in myelin, neurites, and neuroglia. The link between MRI changes and behavioral improvements is inconsistent, likely due to heterogeneous temporal dynamics of plasticity and interindividual variability. Because MRI provides only indirect insight into tissue microstructure, initial studies combine complementary contrasts with multivariate statistics to reduce interpretational ambiguities. High-field imaging, cross-modal approaches such as transcranial magnetic stimulation, and cross-species studies further bridge animal models and human research. Together, these developments refine biologically grounded models of human plasticity and hold promise for translational applications in personalized learning and rehabilitation.

## Introduction

Learning is a core function of the brain, yet the neural changes that support it remain only partly understood. Capturing these experience-dependent alterations is essential for identifying learning potential, personalizing education and therapy, optimizing rehabilitation, and evaluating training interventions across development, aging, and disease.

**Figure 1.**
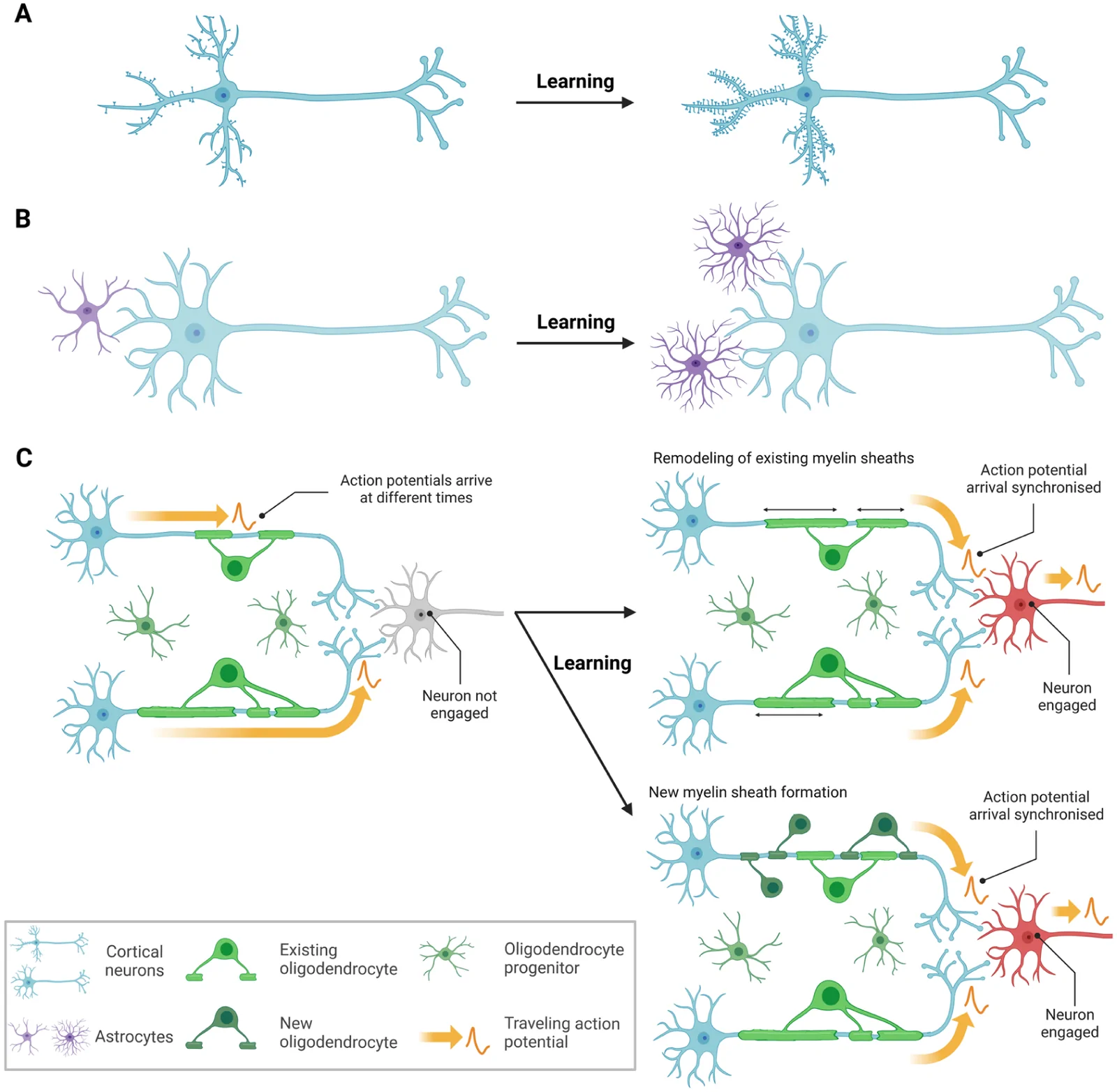
Key mechanisms of learning-induced neuroplasticity in animal models (adapted from Zatorre et al 2012). (A) Dendritic branching and the formation of new synaptic connections. (B) Proliferation and/or hypertrophy of glial cells (here: astrocytes). (C) Adaptation of myelin patterns (thickness, width, distribution) within and between neural circuits shapes conduction velocity, signal timing, and neural synchrony. Myelin plasticity is thought to operate through 2 overlapping processes: the formation of new sheaths and the remodeling of existing ones (Zatorre et al 2012; Bacmeister et al 2022; Mediavilla et al 2022), the latter involving oligodendrocyte proliferation. Additional potential plasticity mechanisms are intentionally omitted. For example, angiogenesis—although observed following aerobic exercise—is considered only minimally relevant to learning (Churchill et al 2002). Likewise, adult neurogenesis in the dentate gyrus, while established, occurs on a limited scale and is therefore unlikely to generate MRI-detectable signal changes (Zatorre et al 2012). Created in BioRender (https://BioRender.com/a81be0i).

Animal research has established cellular mechanisms such as synaptic remodeling, glial dynamics, and adaptive myelination as microstructural substrates of learning (Figure 1), primarily through ex vivo analyses (Zatorre et al 2012). Importantly, studies that combine histology with magnetic resonance imaging (MRI) have shown that structural reorganization can also be detected in vivo (Blumenfeld-Katzir et al 2011; Sagi et al 2012; Sampaio-Baptista et al 2013; Mediavilla et al 2022).

Early human studies used longitudinal T1-weighted MRI (Draganski et al 2004) and diffusion tensor imaging (DTI; Scholz et al 2009) to track learning-related changes over time. While T1-weighted imaging is mainly used to capture macroscopic gray matter properties such as volume, thickness, curvature, or surface area, diffusion MRI probes water mobility shaped by neurite architecture, myelination, membrane properties, and glial organization (Tardif et al 2016; Lerch et al 2017). Because MRI is safe and repeatable, it is particularly well suited for longitudinal studies of human plasticity.

These pioneering studies yielded promising findings and stimulated extensive research (for a review on the early studies, see Zatorre et al 2012), but they also prompted substantive critiques. Concerns centered on the mismatch between MRI’s millimeter-scale resolution and the micrometer-scale architecture of the nervous system, questions of measurement reliability, and methodological limitations such as missing control groups or insufficient statistical rigor (Thomas and Baker 2013). Another major concern raised—the limited biological specificity of conventional MRI (Zatorre et al 2012; Thomas and Baker 2013; Box 1)—restricts both mechanistic insight into learning and clinical translation. The problem becomes particularly pressing in neurologic disease, where microstructural alterations are often selective and frequently precede macroscopic changes detectable with conventional MRI (Lerch et al 2017; Jelescu et al 2020; Dhollander et al 2021; Kraguljac et al 2023). For example, Huntington’s disease is characterized by myelin disruption and axonal degeneration (Casella et al 2020), whereas traumatic brain injury mainly involves diffuse axonal injury (Liang et al 2021), highlighting the need for highly specific biomarkers that can resolve such changes at the relevant biological scale—something conventional MRI alone cannot provide.

**Box 1.** The Limited Biological Specificity of “Conventional” Magnetic Resonance Imaging.

T1-weighted imaging lacks specificity regarding the brain’s cellular architecture. Its signal is constrained by relatively low signal-to-noise ratios, spatial intensity variations arising from radiofrequency field inhomogeneities, and reflects contributions from multiple tissue components, including macromolecules, iron, and water (Sereno et al 2013; Tardif et al 2016; Lerch et al 2017; Weiskopf et al 2021). Although diffusion tensor imaging (DTI) provides orientation-dependent information that is better suited to probing tissue microstructure, it too has notable limitations. Conventional DTI relies on relatively low diffusion-encoding gradients (*b*-values typically ≤1000 s/mm²) and assumes Gaussian water diffusion—an assumption that is violated in restricted geometries such as neurite or glia processes (Jensen et al 2005; Zhang et al 2012; Jelescu et al 2020). In regions with complex tissue architecture, such as voxels containing crossing fibers, DTI-derived metrics conflate microscopic tissue properties with mesoscopic features, such as fiber orientation dispersion (Shemesh 2018; Dhollander et al 2021). As with T1-weighted imaging, the resulting signal reflects a composite of multiple cellular constituents within each voxel and therefore lacks compartment-specific biological interpretability.

MRI is uniquely positioned to meet this challenge. As a multifaceted imaging modality, it can generate a wide range of image contrasts by exploiting intrinsic differences in tissue magnetization properties. Rapid advances in hardware, acquisition strategies, and biophysical modeling have begun to convert this versatility into meaningful progress in biological specificity (Tardif et al 2016; Lerch et al 2017), bringing the field ever closer to an ambitious goal: achieving in vivo—or “virtual”—histology (Weiskopf et al 2021), in which microstructural features can be inferred quantitatively in the living human brain.

This review synthesizes recent studies on learning-related plasticity in humans based on such advanced MRI techniques. We focus on longitudinal studies that include at least 2 MRI measurements acquired before and after a learning or training intervention and that employ relaxometry, magnetization transfer, proton density, or diffusion imaging, often combined with advanced biophysical modeling. We define learning-related plasticity pragmatically as experience-dependent changes in brain microstructure associated with acquiring or refining goal-directed actions (Zatorre et al 2012). This includes domains such as language learning, cognitive training, perceptual learning, motor skill acquisition, and music but excludes more general lifestyle activities such as aerobic exercise.

We begin this review with a basic introduction to the emerging quantitative MRI methodology, as we consider this essential for understanding and contextualizing the empirical findings. These findings will then be presented by domain. Next, we review what these studies reveal about the microstructural correlates of learning in humans across different time scales (i.e., days to weeks), before examining links to behavioral outcomes. Finally, we conclude by reflecting on persistent challenges and future directions.

## Imaging Human Brain Microstructure In Vivo

Studying learning-related plasticity in humans is challenging because many sophisticated experimental methods available in animals are not feasible in human research. Whereas structural and molecular staining of animal tissue can provide submicrometer detail, MRI signals inevitably reflect the aggregate properties of the many tissue components contained within a voxel (Novikov et al 2019; Jelescu et al 2020). To complicate matters further, microstructural plasticity rarely arises from a single biological process (Zatorre et al 2012; Tardif et al 2016; Lerch et al 2017). Consequently, combining advanced MRI techniques to leverage their complementary strengths, as discussed later in this review, appears particularly promising (Cercignani and Bouyagoub 2018).

**Figure 2.**
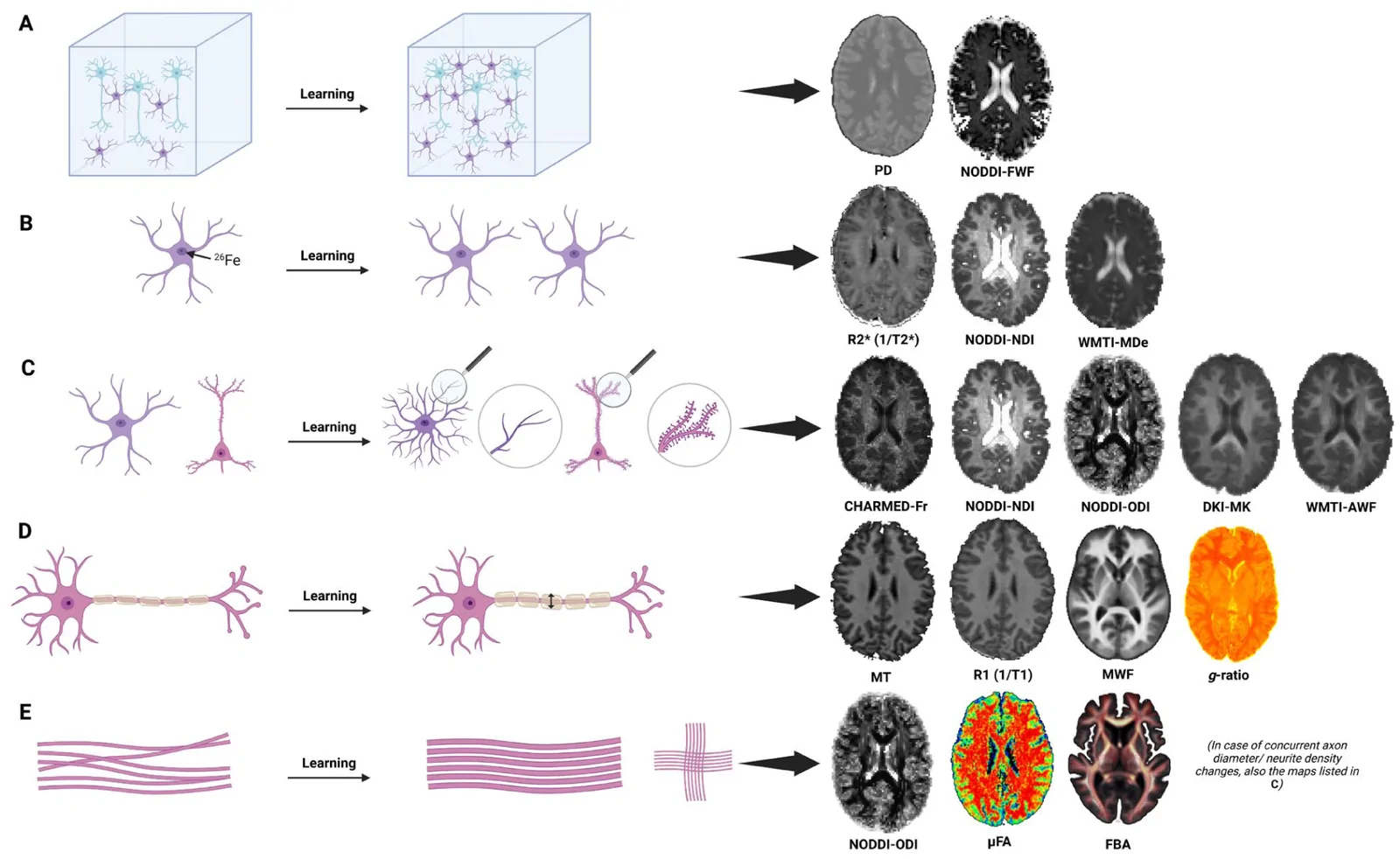
Magnetic resonance imaging (MRI) detection of learning-induced microstructural changes. MRI provides indirect measures of microstructural plasticity, in contrast to histology. Shown are mechanisms potentially detectable with advanced MRI. Although several other MRI metrics—including some depicted—are also sensitive to the depicted mechanisms, only those with the highest relative specificity are shown. (A) Expansion of tissue volume, for example, through cell body proliferation or hypertrophy, leads to decreased water content and reduced free diffusion. (B) Glial proliferation/hypertrophy, affecting R2* through their iron-rich nuclei and giving rise to hindered (extra-axonal) diffusion. In the neurite orientation dispersion and density imaging (NODDI) model, reduced free diffusion (see A), combined with *decreased* neurite density index, may indicate such effects. Future studies will likely employ methods with even higher sensitivity to cell bodies (Palombo et al 2020; Box 2). (C) Confined neuronal (e.g., dendrites) and glial processes cause restricted diffusion. Axons similarly impose diffusion restrictions, but their effects are covered in panel E. Dendritic or synaptic changes might also influence R2* (cf. Azzarito et al 2023; not depicted). (D) Macromolecular content, largely myelin, drives relaxometry and magnetization transfer signals. (E) Training-induced adaptations of axons may involve changes in orientational coherence or thickness, affecting restricted diffusion. Unlike the simplified depiction of a single dominant fiber population shown here, fiber crossings in a voxel are quite common (right). Advanced diffusion metrics—such as μFA, fixel-based analysis (FBA), or NODDI—can cope with these more complex architectures, resolving anisotropic structures aligned in several orientations. Axonal changes likely interact directly with local myelin properties (Zatorre et al 2012), which are omitted here for conceptual clarity. Created in BioRender (https://BioRender.com/lw47kfb). Source of the μFA image in the lower panel: Li et al (2022), CC BY 4.0.

Although in vivo MRI cannot directly quantify specific microstructural properties—such as neuron- or glia-specific markers—microscopic information is assumed to be partially preserved in the MRI signal. MRI uses radiofrequency pulses to manipulate hydrogen nuclei, which emit signals as they relax or interact with their environment (Möller et al 2019; Weiskopf et al 2021). Magnetic field gradients encode the spatial origin of these signals, while tissue-specific properties—such as proton density, relaxation times, molecular interactions, and water diffusion—determine contrast. By adjusting the timing of magnetic field manipulations (pulse sequences), MRI can be tuned to emphasize particular microstructural features, producing relatively specific (“weighted”) maps (Lerch et al 2017; Weiskopf et al 2021). Combined with recent advances in scanner hardware and increasingly sophisticated biophysical modeling and analysis approaches, these developments enable inferences about microstructural characteristics across the whole brain from signals measured at the millimeter scale (Figure 2).

In the following, we briefly summarize key advances in structural neuroimaging that have fueled recent research on human neuroplasticity, providing the conceptual foundation for the empirical results presented later. This overview is not comprehensive; a full review of each method is beyond the scope of this article. Similarly, important issues such as measurement reliability and validation—each warranting detailed discussion in its own right—can only be briefly addressed here, and readers are referred to specialized reviews (Lerch et al 2017; Möller et al 2019; Jelescu et al 2020; Weiskopf et al 2021; Kraguljac et al 2023; Lampinen et al 2023; van der Weijden et al 2023).

### Relaxometry, Magnetization Transfer, and Proton Density

Quantitative MRI measures such as relaxometry, magnetization transfer, and proton density probe the biophysical origins of magnetic resonance (MR) contrast and provide complementary information about water–macromolecule interactions and myelin content (Deoni 2010; Möller et al 2019; Weiskopf et al 2021).

*Relaxometry* imaging is a key technique for probing how water protons interact with their microenvironment. The longitudinal relaxation time (T1) reflects the rate at which excited water proton spins exchange energy with their surrounding molecular environment (“lattice”). T1 shortens in tissues with high macromolecular or paramagnetic content (e.g., proteins, lipids, cell membranes, or iron) and lengthens in regions dominated by free water, such as cerebrospinal fluid or edema (Tardif et al 2016; Lerch et al 2017; Weiskopf et al 2021). The dominant contrast driver appears to be regional myelin content—with a modest iron contribution—such that T1 maps closely resemble cortical myeloarchitecture (Sereno et al 2013).

The transverse relaxation time (T2) reflects the loss of phase coherence among water protons due to local molecular interactions, while T2* captures additional signal decay arising from local magnetic field variations, for example, those induced by paramagnetic iron (Tardif et al 2016; Weiskopf et al 2021). Although iron is not a distinct cellular compartment, its quantitative assessment provides valuable insight because of its presence in neuroglia and myelinated fibers (Deoni 2010; Möller et al 2019). As iron is thought to be consumed during synaptogenesis and restored during synaptic pruning, R2* might also provide information relevant to synaptic plasticity (Azzarito et al 2023).

Here, relaxation times (T1, T2, T2*) and the corresponding rates (R1 = 1/T1, R2 = 1/T2, R2* = 1/T2*) are used interchangeably.

Multicomponent relaxometry (MCR) exploits the differing relaxation behaviors of fast-decaying water trapped between the myelin bilayers (short T2) and the more slowly relaxing intra- and extracellular water. The resulting myelin water fraction (MWF) serves as a sensitive, histologically validated marker of myelin content (Deoni 2010; van der Weijden et al 2023).

Magnetization transfer (MT) imaging complements relaxometry by selectively saturating protons bound to macromolecules, such as those in myelin. This saturation transfers to nearby free water protons and reduces the measured signal, so stronger MT effects indicate higher macromolecular and myelin content (Lerch et al 2017; Weiskopf et al 2021; van der Weijden et al 2023). In contrast, the effective proton density (PD) reflects MR-visible water content that decreases with increasing macromolecular content.

Because relaxometry, MT, and PD mapping are each differentially sensitive to specific tissue properties, their combined use offers a more nuanced and biologically interpretable characterization of brain microstructure. The multiparameter mapping (MPM) framework (Helms et al 2008) integrates R1, R2*, MT, and PD mapping within a single, time-efficient (~20 min) protocol at a 1-mm isotropic resolution and has demonstrated low intersite bias in a multicenter study (Weiskopf et al 2013).

### Advanced Diffusion Imaging: Signal Representations and Biophysical Models

DTI has long been the workhorse for assessing microstructural plasticity and performs well in white matter regions with approximately parallel fiber bundles. However, DTI is limited in its ability to probe more complex tissue architectures—such as regions with crossing, fanning, or kissing fibers—or gray matter, where each voxel typically contains multiple microscopic tissue environments that differ in size and anisotropy (Zhang et al 2012; Novikov et al 2019; Jelescu et al 2020; Figure 2).

Advanced diffusion imaging approaches, which require high-performance gradient systems, are designed to address these limitations. These methods typically sample diffusion space in at least 60—and often hundreds of—gradient directions, a strategy known as high-angular-resolution diffusion imaging (HARDI). They also employ high-diffusion-weighting strengths (*b*-values ≥2000 s/mm²) and multiple nonzero *b*-values (multishell acquisitions). Such acquisitions enable probing of non-Gaussian diffusion, increasing sensitivity to neurites and complex microstructural geometries (Fukutomi et al 2018; Jelescu et al 2020; Reveley et al 2022). Depending on the analysis framework, they may further disentangle diffusion anisotropy in cellular tissue compartments from more mesoscopic effects due to orientational dispersion of fibers (Shemesh 2018; Lampinen et al 2023).

To derive quantitatively and biologically meaningful measures of brain tissue, the diffusion signal must be described or modeled in a mathematically and biophysically plausible manner. *Signal representations* provide statistical descriptions of diffusion attenuation, whereas *biophysical multicompartment models* incorporate assumptions about underlying tissue properties (Novikov et al 2019; Jelescu et al 2020; Lampinen et al 2023; Box 2). These models can yield more specific insights into microstructural alterations, such as neuritic changes in gray and white matter, provided that the underlying modeling assumptions are valid (see next section).

**Box 2.** Signal Representations and Biophysical Models of Diffusion.

**Figure 3.**
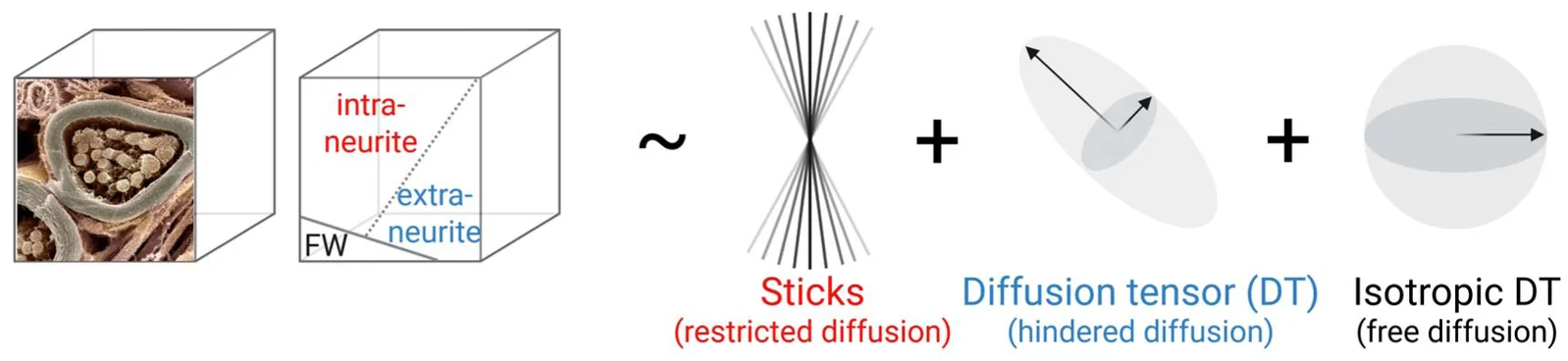
Neurite orientation dispersion and density imaging (NODDI; Zhang et al 2012) as an example implementation of the “standard model” of diffusion in biological tissue. NODDI, like other models in this family, assumes that the diffusion magnetic resonance imaging signal reflects a weighted sum of water diffusing in distinct tissue compartments. Cerebrospinal fluid exhibits free diffusion and is modeled as an isotropic Gaussian “ball,” giving rise to the free water fraction (FWF). Neural tissue is represented by 2 compartments: 1) intra-neurite water within axons, dendrites, and glial processes, producing restricted, non-Gaussian diffusion modeled as “sticks,” whose angular dispersion is described by a Watson distribution and quantified by the orientation dispersion index (ODI), and 2) the extra-neurite space, characterized by hindered diffusion and modeled with an anisotropic tensor. The neurite density index (NDI) represents the relative contribution of the intra-neurite compartment to the tissue signal. Thus, increases or decreases in NDI (assuming stable free water fraction) indicate shifts in the balance between intra- and extra-neurite signal fractions. Figure inspired by Kraguljac et al (2023). Created in BioRender (https://BioRender.com/c8mlpk5). Source of the myelinated nerve fiber image: David Furness. Wellcome Collection. Licensed under CC BY 4.0 (https://creativecommons.org/licenses/by/4.0/)

Water diffusion in brain tissue deviates from an ideal Gaussian pattern due to microstructural barriers. Diffusion kurtosis imaging (DKI) extends diffusion tensor imaging (DTI) by quantifying this non-Gaussianity, providing measures of intravoxel diffusional heterogeneity (Jensen et al 2005). Higher kurtosis generally reflects more restricted diffusion, for example, from cell membranes or densely packed neurites. While DKI is more sensitive to microstructural complexity than DTI, its measurements are not specific to particular biological sources and can be influenced by macroscopic voxel heterogeneity, such as crossing fibers or partial volume effects. One approach specifically designed to resolve crossing fibers is constrained spherical deconvolution (CSD). Using high-angular-resolution diffusion imaging data, CSD models the diffusion signal in each voxel as a combination of contributions from fibers with different orientations (Raffelt et al 2017; Dhollander et al 2021). By applying a fiber response function—the expected signal from a single, coherently oriented fiber bundle—CSD estimates the fiber orientation distribution function, enabling accurate characterization even in regions with complex fiber architecture. Building on CSD, fixel-based analysis (FBA) quantifies individual fiber populations within a voxel (“fixels”), rather than collapsing multiple fiber populations into a single voxel-averaged metric (Raffelt et al 2017; Dhollander et al 2021). For each fixel, FBA estimates 3 parameters: fiber density (FD), reflecting intra-axonal volume; fiber cross section (FC), capturing macroscopic bundle morphology; and their combined measure, fiber density and cross section (FDC). Increases in these metrics generally indicate greater intra-axonal volume (e.g., increased axon number or diameter), larger bundle size, or overall higher fiber capacity, respectively, for FD, FC, and FDC. Other approaches to resolving complex fiber geometries, such as quantitative anisotropy (QA) based on generalized q-sampling imaging (GQI) or diffusion spectrum imaging (DSI), also exist (Yeh et al 2010); however, to our knowledge, these methods have not yet been applied in longitudinal studies of training-induced neuroplasticity. Another approach to probing complex microstructure moves beyond the conventional Stejskal–Tanner acquisition framework (Lampinen et al 2023), which encodes diffusion along a single direction. One implementation of so-called multidimensional diffusion encoding methods is double-diffusion encoding (DDE), in which 2 diffusion encoding periods are applied prior to image readout, separated by a mixing time (Shemesh 2018). This approach enables estimation of parameters such as microscopic fractional anisotropy (μFA), reflecting local anisotropy independent of fiber orientation (Shemesh 2018). Unlike conventional FA, μFA remains high in regions with complex fiber crossings when bundles are intact, providing a relatively unbiased measure of local tissue architecture (Li et al 2022). The aforementioned methodological approaches do not rely on strong assumptions about microstructural tissue composition. In contrast, biophysical models typically parametrize the measured diffusion MRI signal as a weighted sum of 2 or more nonexchanging compartments, defined by their intracompartment diffusivities and relative sizes (Novikov et al 2019; Jelescu et al 2020; Weiskopf et al 2021; Lampinen et al 2023). These models aim to distinguish hindered, yet approximately Gaussian, diffusion in the extraneurite space (e.g., cell bodies and extracellular matrix) from restricted, non-Gaussian diffusion within more confined environments, such as cell processes (axons, dendrites, and neuroglial processes). These processes are often modeled as a collection of “sticks” (cylinders with zero radius) with a characteristic orientation dispersion function (Novikov et al 2019), embedded within the extra-axonal space. This so-called standard model (Novikov et al 2019) branches into several specific implementations, including white matter tract integrity (WMTI; Fieremans et al 2011), the composite hindered and restricted model of diffusion (CHARMED; Assaf and Basser 2005), and neurite orientation dispersion and density imaging (NODDI; Zhang et al 2012; Figure 3), each introducing its own assumptions.
Some characteristics and features of the models mentioned above should be noted. NODDI, in particular, enables estimation of a free water fraction, which is used to correct other model parameters and allows quantification of the orientation dispersion index (ODI) of the neurite compartment (Figure 3). Unlike FBA, however, NODDI does not explicitly model multiple fiber populations but interprets crossing configurations as a single fiber population with high dispersion (Dhollander et al 2021). WMTI is optimized for highly aligned white matter and limited to that domain (Fieremans et al 2011; Huber et al 2021), whereas CHARMED and NODDI have also been applied to study microstructural plasticity in gray matter. For NODDI, specific adaptations of the model parameters have been proposed for gray matter, with the optimized neurite density index (NDI) related to cortical myelin content and the ODI characterizing cortical neurite microarchitecture (Fukutomi et al 2018).

### Biophysical Models—A Note of Caution

Although advanced MRI techniques show encouraging reproducibility and growing validation against histologic benchmarks (Weiskopf et al 2013; Lerch et al 2017; Jelescu et al 2020; Kraguljac et al 2023; van der Weijden et al 2023), they remain fundamentally indirect probes of tissue microstructure. Biological interpretation therefore depends on biophysical models that introduce simplifying assumptions (Figure 3). For example, the multicompartment models of diffusion introduced above represent neurites as zero-radius sticks with maximally anisotropic diffusion, impose relationships or hard constraints on intra- and extra-axonal diffusion properties, or neglect intercompartmental relaxation differences and water exchange. These assumptions are still under debate; for instance, the stick approximation may be problematic in gray matter dendrites, and differences in relaxation between tissue classes confound the estimated volume fractions (Novikov et al 2019; Jelescu et al 2020; Lampinen et al 2023). Moreover, multicompartment diffusion models are inherently degenerate: different combinations of parameters can explain the same signal, potentially leading to interpretational ambiguities (Jelescu et al 2020). Myelin-sensitive imaging approaches based on relaxation or magnetization transfer rely on their own simplifying assumptions discussed elsewhere (Möller et al 2019).

Potential remedies and future directions in data acquisition and modeling are summarized in Box 3. These approaches, however, typically require nonstandard pulse sequences and/or extended acquisition times, which currently limit their broader adoption.

**Box 3.** Advances in Data Acquisition and Modeling to Improve Biological Specificity.

Recent methodological developments aim to provide more independent and information-rich measurements, thereby reducing reliance on strong model assumptions. Examples include multi-*b*-shape acquisitions that extend the conventional Stejskal–Tanner framework (Shemesh 2018; Lampinen et al 2023; see also Box 2, “multidimensional diffusion encoding”) and joint diffusion–relaxation protocols. Multi-echo diffusion acquisitions, for instance, seek to mitigate T2-related bias and improve estimation of compartmental volume fractions (Gong et al 2020). Other joint diffusion–relaxation approaches combine diffusion magnetic resonance imaging (MRI) with myelin-sensitive contrasts (e.g., proton density or magnetization transfer) to derive composite metrics such as the aggregate axonal *g*-ratio—a biophysically interpretable index of relative myelination that strongly influences axonal conductivity (Stikov et al 2015). Complementary advances in biophysical modeling aim to better represent cellular contributors to the MRI signal that remain insufficiently captured by current frameworks. Neuroglial changes, for example, are thought to play a significant role in learning, as demonstrated in animal studies (Blumenfeld-Katzir et al 2011; Figure 1). Yet the biophysical models of diffusion introduced above aggregate all tissue signal contributions outside elongated cellular structures (“neurites”) into a single extra-neurite compartment. In contrast, soma and neurite density imaging by diffusion MRI (SANDI) differentiates soma-related from extracellular water contributions, building on high-gradient diffusion evidence that somata produce a distinct signal signature (Palombo et al 2020). Although empirical training studies using SANDI remain scarce, initial applications (e.g., Griffa et al 2025) and ongoing work (e.g., Ioakeimidis et al 2024) suggest promising sensitivity to gray matter microstructural change. SANDI therefore represents a compelling avenue for future intervention studies, although its relevance for training-induced plasticity remains to be established.

## Empirical Results from Longitudinal Studies

Having introduced several key concepts of advanced microstructural imaging using MRI in humans, we now review empirical evidence from longitudinal studies, organized into broad thematic sections: motor training, cognitive training, language, perception, and music. Inevitably, some behavioral paradigms (e.g., Braille learning) do not fit unambiguously into a single category. In the following, we focus on the most relevant findings, which we will discuss in greater depth later.

### Motor Domain

#### Relaxometry, magnetization transfer, and proton density

Using multicomponent relaxometry, Lakhani et al (2016) reported significant increases in the MWF in the left intraparietal and parieto-occipital sulci within participants who practiced an intensive right arm visuomotor task over 4 weeks (10,000 repetitions). Similarly, Kirby et al (2022) observed within-group MWF increases in the right corticospinal tract after 2 weeks of training on a visuomotor tracking task requiring complex cursor control. In a rehabilitation setting, Faw et al (2021) demonstrated that 12 weeks of downhill treadmill training in individuals with chronic incomplete spinal cord injury led to increased MWF in cortical regions implicated in motor learning (postcentral gyrus, precuneus) and in the ventral funiculi of the spinal cord relative to controls.

Azzarito et al (2023) examined microstructural trajectories during 4 weeks of training on a dance and rhythm game (StepMania) using the MPM protocol. Across motor (cerebellum, corticospinal tract) and limbic regions (hippocampus), nonmonotonic temporal patterns emerged, with initial decreases followed by later increases in between-group differences (MT, R1, R2*). The trained group also showed stronger negative linear R2* changes in the left corona radiata and right anterior cerebellum compared with controls. Temporal-lag analyses further revealed that early motor system changes predicted later hippocampal plasticity, suggesting a sequential engagement of distinct learning systems (Azzarito et al 2023).

#### Diffusion imaging

In young, healthy adults, Tavor et al (2013) demonstrated that participants who learned a car-racing task for just 2 h exhibited an increase in the volume fraction of the restricted compartment (Fr) of the composite hindered and restricted model of diffusion (CHARMED) model in the left hippocampus and right parahippocampal gyrus. Consistent with these findings, Stee et al (2023) reported increases in NDI in subcortical regions, as assessed with neurite orientation dispersion and density imaging (NODDI), including the hippocampus and putamen, after only 1 h of motor sequence learning. They also observed decreased free water fraction (FWF) in the cerebellum, hippocampus, thalamus, and caudate, as well as an increased orientation dispersion index (ODI) in the putamen. In addition, widespread cortical NDI increases overlapped with FWF decreases, whereas ODI changes were smaller and region-specific (Stee et al 2023).

In pubescent patients with traumatic brain injury, Liang et al (2021) found that 8 weeks of home-based balance training led to significant increases in fiber cross section (FC) and fiber density–cross section (FDC) within left sensorimotor tracts, as assessed using fixel-based analysis (FBA), without accompanying changes in fiber density (FD). This pattern was interpreted as macrostructural restoration of damaged pathways. The sensitivity of FBA to such macrostructural white matter changes is further supported by Koschutnig et al (2024), who showed that 3 weeks of slackline training increased FC in sensorimotor-related tracts (e.g., superior longitudinal fasciculi, corticospinal tract), accompanied by decreases in NODDI-derived NDI and ODI in overlapping regions—effects not observed in controls. In contrast, Ueta et al (2022) and Mizuguchi et al (2019) did not report significant FBA changes after a single slackline session or after 2 days of practice on a complex whole-body serial reaction time task, respectively.

#### Multicontrast studies

In young healthy adults, 4 weeks of dynamic whole-body balance training were associated with changes in NODDI and MPM metrics. NODDI-ODI increased across widespread motor regions (Lehmann et al 2023). Concurrently, MPM metrics revealed an R2* increase in the right fusiform gyrus, a trend toward reduced R2* in the left hippocampus, and decreased MT in the left medial frontal cortex relative to a nontraining control period (Aye et al 2025). In parallel, motor-related white matter tracts also exhibited changes, most prominently reflected in the aggregate *g*-ratio (Aye et al 2026).

In patients with Huntington’s disease (HD), Casella et al (2020) examined the effects of a 2-month drumming intervention using quantitative T1, MT, and CHARMED imaging. Patients with HD showed a significant posttraining increase in MT relative to matched healthy controls, particularly in the corpus callosum and the right striato–supplementary motor loop.

### Cognitive and Spatial Navigation Training

#### Diffusion imaging

In what is, to our knowledge, the only intervention study using double diffusion encoding (DDE), Li et al (2022) reported a decrease in microscopic fractional anisotropy (μFA) following 4 weeks of attention and working memory training—an effect not detected by conventional DTI metrics. Villemonteix et al (2023) examined microstructural changes after a single 40-min navigation task in a virtual environment and observed a widespread decrease in the NODDI-FWF—consistent with local increases in tissue volume—across extensive cortical and subcortical regions. Sleep deprivation during the offline consolidation period further amplified changes, yielding greater increases in FWF in the right caudate and higher NDI in both the right caudate and left hippocampus compared with regular sleep. In contrast, Verhelst et al (2019) found no white matter changes in adolescents with traumatic brain injury following 8 weeks of computerized cognitive training using FBA, and McPhee et al (2021) reported that *Bacopa monnieri* supplementation did not alter NODDI measures in older adults undergoing cognitive training.

#### Multicontrast studies

In young healthy adults, Metzler-Baddeley et al (2017) examined the effects of adaptive versus nonadaptive working memory training over 40 sessions across 8 weeks. Using principal component analysis (PCA) to jointly assess changes in MWF, R1, and CHARMED metrics across multiple regions, they found that adaptive training produced significant alterations in a latent microstructural component relative to the nonadaptive group. These changes included increased R1, restricted volume fraction (CHARMED-Fr), and fractional anisotropy (DTI), together with reduced radial diffusivity, with the strongest loadings observed in the right superior longitudinal fasciculus and left parahippocampal cingulum.

### Language, Braille, and Literacy

#### Relaxometry, magnetization transfer, and proton density

Economou et al (2023) showed that a 12-week tablet-based literacy intervention increased MWF in kindergarteners at risk for dyslexia—specifically in left hemispheric reading tracts and right ventral pathways—revealing significant interaction effects relative to both active and inactive control groups. Similarly, Matuszewski et al (2021) reported within-group R1 increases after 8 months of Braille training, particularly in the frontal lobes and ventral occipitotemporal cortex, a key region for written language. These structural changes emerged later in training, following earlier functional changes observed with task-based functional MRI (fMRI).

#### Diffusion imaging

Using DKI, Vukovic et al (2021) found that a single 40-min immersive vocabulary session induced rapid microstructural changes in core semantic hubs: mean kurtosis (DKI-MK) increased in the left anterior temporal lobe, whereas mean diffusivity (DTI-MD) decreased in the angular gyrus. These effects differed significantly from those in a group receiving disruptive transcranial magnetic stimulation (TMS) over M1, a region implicated in action-language processing. At a 24-h follow-up, both groups showed decreased DTI-MD and increased DTI-MK in the left caudate and hippocampus.

#### Multicontrast studies

In preadolescent children with reading difficulties, Huber et al (2021) observed widespread decreasing trajectories in DTI-MD during an intensive 8-week reading intervention, spanning, among others, the arcuate, inferior longitudinal, and inferior fronto-occipital fasciculi. Parallel decreases appeared in extra-axonal mean diffusivity from white matter tract integrity (WMTI), and neither effect was present in age-matched controls. Unlike the myelin-related findings of Economou et al (2023) and Matuszewski et al (2021), the authors observed no accompanying changes in R1. Likewise, the neurite-related marker axonal water fraction from WMTI remained unchanged (Huber et al 2021).

### Perception and Visual Learning

#### Relaxometry, magnetization transfer, and proton density

Using the MPM protocol, Ziminski et al (2023) investigated the effects of 3 days of practice on a perceptual decision-making task. Training led to increased MT values in subcortical regions, including the pulvinar and hippocampus, whereas MT remained stable during a preceding control period without training.

#### Diffusion imaging

Alharshan, Aloufi et al (2025) examined the effects of 6 weeks of home-based eye movement training using DKI and DTI. Within the training group, reductions in mean, radial, and axial kurtosis (DKI-MK, DKI-RK, DKI-AK) and mean diffusivity (DTI-MD) were detected in task-relevant visual areas—particularly the cuneus and pericalcarine cortex—with kurtosis measures showing greater sensitivity to training-related changes than tensor metrics in the pericalcarine region. In a related study, Alwashmi et al (2025) investigated 4 weeks of reality-based training designed to promote adaptation to novel audiovisual stimulus configurations. They interpreted their results as evidence that DTI and DKI offer complementary information, with within-group DKI decreases (MK, RK) primarily localized to task-relevant regions (primary visual and auditory cortices) and their interconnecting white matter pathways, including the inferior longitudinal and fronto-occipital fasciculi.

### Music

#### Diffusion imaging

Jünemann et al (2022) conducted a 6-month randomized controlled trial to test whether piano training could mitigate age-related white matter decline in healthy older adults. Using FBA, they found that participants who received piano training maintained fiber density (FD) in the fornix, whereas the active control group—who engaged in music listening without motor practice—showed significant FD reductions.

## Time Courses and Potential Mechanisms

Having presented the relevant longitudinal studies by domain, we now turn to the question of time scales. For synthesis, we distinguish between short-term (≤1 week) and longer-term (≥3 weeks) effects, recognizing that these categories are pragmatic rather than biologically discrete. The key conclusions from the following sections are summarized in Table 1.

**Table 1.** Summary of the Main Findings on Microstructural Plasticity Associated with Learning in Humans across Different Time Scales.

	Short Interventions (<1 Week)	Longer Interventions (>3 Weeks)		
		Myelin	Cell Process Remodeling	Extra-axonal Mechanisms
MRI signatures	↑ Restricted diffusion (e.g., DKI-MK, CHARMED-Fr, NODDI-NDI); ↑ local tissue fraction/↓ free water (e.g., NODDI-FWF), partial persistence across days with reinstatement after relearning	↑ Myelin-sensitive markers (MWF, MT, R1, *g*-ratio), occasional early decreases or nonmonotonic trajectories	Restricted diffusion and orientation dispersion (e.g., NODDI-ODI, DKI-MK, DDE-μFA), R2* alterations	↑ tissue expansion (FBA-FC, NODDI-FWF); kurtosis and fiber density alterations
Predominant anatomical loci	Primarily cortical and subcortical gray matter, especially hippocampus; no consistent white matter effects	Task-relevant gray and white matter	Task-relevant cortical and subcortical gray matter	Task-relevant white matter
Candidate mechanisms	Early dendritic remodeling, glial hypertrophy or proliferation, glial process expansion, extracellular matrix remodeling	Myelin-related mechanisms (e.g., changes in sheath thickness, internode length, node of Ranvier properties)	Dendritic elaboration or pruning, glial process reorganization	Glial hypertrophy or gliogenesis, extracellular matrix remodeling, axonal or glial process reorganization

See text for further details and references. CHARMED, composite hindered and restricted model of diffusion; DDE, double-diffusion encoding; DKI, diffusion kurtosis imaging; FBA, fixel-based analysis; FC, fiber cross section; Fr, volume fraction of the restricted compartment; FWF, free water fraction; MK, mean kurtosis; MRI, magnetic resonance imaging; MT, magnetization transfer; MWF, myelin water fraction; NDI, neurite density index; NODDI, neurite orientation dispersion and density imaging; ODI, orientation dispersion index; μFA, microscopic fractional anisotropy.

### Short Intervention Periods (<1 Week)

The findings reviewed above demonstrate that microstructural reorganization in response to learning can emerge remarkably rapidly—sometimes within minutes. Two consistent patterns characterize studies employing very brief intervention periods (<1 week).

First, all such studies find training-induced changes in cortical and, more prominently, subcortical gray matter microstructure, whereas white matter alterations have not been observed. Despite domain-specific differences in the regions involved, one structure stands out: the hippocampus. Rapid hippocampal plasticity appears across motor (Tavor et al 2013; Stee et al 2023), cognitive (Villemonteix et al 2023), perceptual (Ziminski et al 2023), and language learning (Vukovic et al 2021), supporting an early, domain-general role in learning (Frankland and Bontempi 2005).

Second, across domains, the predominant microstructural signatures consist of increases in restricted diffusion (Tavor et al 2013; Vukovic et al 2021; Stee et al 2023; Villemonteix et al 2023) and increases in local tissue fraction (Stee et al 2023; Villemonteix et al 2023). These patterns align with 2 potentially complementary mechanisms of rapid plasticity proposed in animal work—summarized by Zatorre et al (2012) and discussed early on by Tavor et al (2013). Enhanced restricted diffusion may reflect mechanisms such as dendritic growth or expansion of glial processes, whereas increases in tissue fraction relative to free water could arise from glial cell hypertrophy or proliferation (Figure 2). Theoretically, neurogenesis in the dentate gyrus could play a role as well, but given the low number of newly generated cells, it is unlikely to have a profound impact on MRI-detectable microstructural change (Zatorre et al 2012).

Although increased restricted diffusion in the gray matter is often interpreted in the context of neurite morphology (e.g., Reveley et al 2022), evidence from Fukutomi et al (2018) indicates that it can also reflect myelin-related mechanisms. That only Ziminski et al (2023) detected changes in a relatively myelin-specific metric (magnetization transfer) during brief training may therefore reflect methodological choices, rather than implying that early-stage myelin plasticity does not occur in humans.

Regarding the time course of early learning, microstructural changes from the first practice session persist only partly by the next day(s), but relearning appears to reinstate them, usually with reduced magnitude and tighter spatial focus (Tavor et al 2013; Stee et al 2023; Villemonteix et al 2023).

### Longer Intervention Periods (≥3 Weeks)

#### Myelin-sensitive metrics in gray matter

With longer intervention periods, myelin remodeling appears to play a more prominent role than in shorter training. In both cortical and subcortical gray matter, myelin-sensitive metrics frequently show training-related changes in task-relevant regions during motor (Lakhani et al 2016; Faw et al 2021; Azzarito et al 2023; Aye et al 2025) and language learning (Matuszewski et al 2021), consistent with evidence from animal models indicating an important role of cortical myelination in learning (Mediavilla et al 2022). Comparable patterns are observed in task-relevant white matter pathways, which reorganize in response to motor (Casella et al 2020; Kirby et al 2022; Aye et al 2026), language (Economou et al 2023), and cognitive training (Metzler-Baddeley et al 2017).

Across these studies, increases in MWF, MT, and R1, as well as reductions in the aggregate *g*-ratio, are most commonly reported, suggesting enhanced myelination as a plausible underlying mechanism (Sereno et al 2013; van der Weijden et al 2023). However, some studies also document decreases in these metrics following training (Aye et al 2025) or transient reductions early in the intervention (Azzarito et al 2023), highlighting complex temporal dynamics in myelin plasticity—similar to patterns observed in animal models (Bacmeister et al 2022; Mediavilla et al 2022; Figure 1).

#### Restricted diffusion in the gray matter

**Figure 4.**
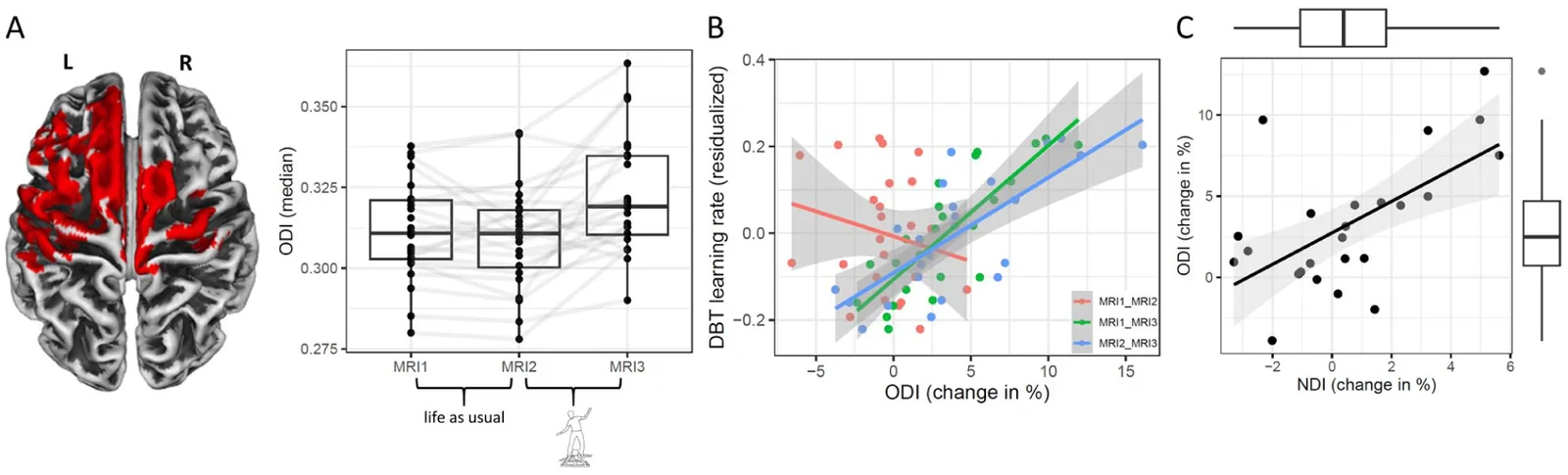
Exemplary findings from the balance-learning study by Lehmann et al (2023). (A) A 4-week training intervention to acquire a complex whole-body balance skill was preceded by an equally long no-training control period. A significant increase in neurite orientation dispersion and density imaging’s orientation dispersion index (ODI) was demonstrated across widespread motor-related cortical areas, but only during the training period. (B) Moreover, ODI increases during training correlated significantly with balance-learning performance, whereas no such relationship was found in the control period. This pattern was interpreted as evidence for behaviorally relevant increases in restricted diffusion, potentially driven by dendritic remodeling. (C) Further support came from the observation that ODI changes covaried with changes in the neurite density index (NDI), but not with changes in free water fraction (FWF) or the myelin-sensitive magnetization transfer metric (neither shown).

Another relatively consistent pattern of microstructural plasticity emerges in task-relevant cortical and subcortical gray matter. Here, changes in restricted diffusion suggest modifications in neurite tissue or its organizational complexity (Fukutomi et al 2018; Reveley et al 2022), as well as remodeling of glial processes (Tavor et al 2013; Figure 2). As with myelin-related measures, however, the directionality of these diffusion-based changes is not uniform. For example, following complex balance training, we observed increases in NODDI-ODI across distributed cortical motor networks (Lehmann et al 2023), which might reflect dendritic process elaboration. These changes seemed to be relatively unaffected by cortical myelin, since colocalized MT was unchanged (Figure 4). Others have interpreted decreases in microscopic fractional anisotropy (μFA) after cognitive training as synaptic pruning, including reductions in dendritic branching (Li et al 2022). Similarly, decreases in restricted diffusion reported in DKI studies of perceptual learning (Alharshan et al 2025; Alwashmi et al 2025) might reflect pruning-like mechanisms. Relatedly, gray matter R2* changes following balance training (Aye et al 2025) might also point toward reorganization at the synaptic level, given that iron is thought to be utilized during synaptogenesis and restored during pruning (as discussed by Azzarito et al 2023). Alternatively, such R2* alterations may reflect changes in iron-rich neuroglial populations (Deoni 2010; Möller et al 2019).

#### White matter—beyond myelin

In white matter, while changes potentially associated with myelin remodeling have frequently been observed (see above), other microstructural plasticity mechanisms show less consistency. Alterations in diffusion kurtosis (Alharshan et al 2025; Alwashmi et al 2025), CHARMED’s restricted volume fraction (Metzler-Baddeley et al 2017), or FBA’s apparent fiber density (Jünemann et al 2022) suggest modifications in restricted environments, such as axons or glial processes, as well as changes in their organizational complexity (Figure 2). Increases in fiber bundle cross-section have been reported following balance training (Koschutnig et al 2024; Liang et al 2021), but without corresponding changes in fiber density.

The aforementioned slackline study by Koschutnig et al (2024) warrants further discussion, as it reported a larger FBA-based fiber-bundle cross section in combination with reduced neurite density (NODDI-NDI) and orientation dispersion (NODDI-ODI). This pattern suggests that expansion of intravoxel tissue—driven by extra-neurite mechanisms—may have contributed to the observed changes (Figure 3). Specifically, even if the absolute amount of local neurite tissue remained unchanged, mechanisms such as glial hypertrophy, gliogenesis, or extracellular matrix remodeling could lead to a relative decrease in NDI while simultaneously increasing the overall tract size, as reflected in FBA-FC. Notably, in a similar balance-training paradigm, Aye et al (2026) observed a comparable pattern in the anterior thalamic radiation: a reduction in the NODDI-FWF (suggesting increased tissue occupancy), combined with a tendency toward lower NDI and largely unchanged myelin-sensitive metrics, is more consistent with extra-neurite or glia-related mechanisms than with neurite loss. Supporting this interpretation, Huber et al (2021) demonstrated that white matter plasticity was predominantly driven by extra-axonal changes (WMTI-MDe), without detectable alterations in myelin- or neurite-sensitive markers.

Taken together, these studies indicate that myelin-related plasticity, changes in restricted tissue compartments (e.g., axons or glial processes), and extra-axonal mechanisms (such as cell bodies or extracellular matrix remodeling) can each occur in white matter, consistent with theoretical positions in the literature and findings from animal studies (Zatorre et al 2012; Tardif et al 2016), either with one mechanism predominating or with multiple processes acting in parallel. With respect to changes in restricted tissue compartments, an important caveat should be noted. When myelin-sensitive measurements are not acquired concurrently—or when changes in a metric sensitive to restricted diffusion coexpress with changes in a myelin-sensitive metric (as in Metzler-Baddeley et al 2017)—interpretation becomes inherently ambiguous. Given the well-documented sensitivity of restricted diffusion metrics to myelin (Fukutomi et al 2018; Jelescu et al 2020; Reveley et al 2022), it remains unclear whether such findings reflect primary myelin-related mechanisms or mixed contributions from both myelin and neurite mechanisms.

#### Cross-tissue plasticity

The findings summarized above suggest that learning periods longer than a few weeks predominantly induce synaptic changes in gray matter, whereas myelin plasticity appears to be a consistent feature of white matter. This pattern aligns with basic research showing that synaptic plasticity is closely coordinated with adjustments in axonal conduction velocity, ensuring the timely arrival of action potentials at key network nodes (Zatorre et al 2012; Bacmeister et al 2022; Figure 1).

Although only a few studies have examined both processes within the same sample, emerging evidence supports such coordinated gray–white matter adaptations in humans. For example, Azzarito et al (2023) and Emmenegger et al (2024) report converging patterns consistent with this framework. In the corticospinal tract, colocalized changes in MT together with reductions in radial diffusivity point to myelin-related processes. In the cerebellum, colocalized decreases in R2* and MD more likely reflect synaptic or glial mechanisms (Azzarito et al. 2023; Deoni 2010). Similarly, a recent balance-training study reported correlated gray–white matter adaptations interpreted as predominantly synaptic in cortex (Lehmann et al 2023) and myelin-related in white matter tracts (Aye et al 2026).

### Behavioral Relevance

If microstructural plasticity contributes to improved performance on a learned task, neural changes would be expected to scale with behavioral gains (Thomas and Baker 2013). Across the studies reviewed here, approximately 35% report significant brain–behavior correlations (counting each sample only once). In contrast, 27% did not test or report such associations, and 38% explicitly tested for them but found no significant relationship. Thus, while behaviorally relevant microstructural changes are reported, they are far from ubiquitous.

The significant associations span motor, cognitive, perceptual, and musical learning domains (Table 2). Strikingly, most reported correlations concern gray matter microstructure, whereas only 2 studies identified white matter associations. Moreover, the direction of the correlation can sometimes be unexpected. For instance, both Lakhani et al (2016) and Ziminski et al (2023) observed increases in myelin-sensitive metrics in gray matter at the group mean or interaction level. However, in both studies, the correlation patterns suggested that these increases were associated with lower learning rates. One possible interpretation is that individuals with more efficient baseline circuitry require smaller microstructural adjustments to optimize conduction properties in task-relevant networks. Alternatively, these patterns may reflect factors such as baseline differences in behavior or nonlinear relationships between myelin remodeling and behavioral improvement. In white matter, Aye et al (2026) report that a training-related increase in relative myelination, inferred mainly based on the aggregate *g*-ratio, was positively correlated with learning success. Together, these findings caution against interpreting training-related changes, especially in myelin-sensitive MRI metrics, as uniformly beneficial or detrimental, emphasizing the complex and context-dependent nature of myelin remodeling (Mediavilla et al 2022; Figure 1), as well as the possible influence of moderating variables such as baseline brain status or prior skill level.

**Table 2.** Overview of Studies Reporting Significant Changes between Advanced MRI-Derived Microstructural Measures and Learning.

Study	Paradigm	Type of correlation	Metrics
Lehmann et al 2023; Aye et al 2025; Aye et al 2026	Motor—balance skill learning	Correlated change	GM: NODDI-ODI, R2* WM: multivariate analysis, *g*-ratio as one of the main drivers
Azzarito et al 2023	Motor—dance and rhythm game	Correlated change	GM: R2*
Lakhani et al 2016	Motor—visuomotor skill	Correlated change	GM: MWF
Rakowska et al 2025	Motor—serial reaction time task	Correlated change	GM: CHARMED-Fr
Li et al 2022	Cognitive—attention and working memory training	Correlated change	GM: DDE-μFA
Matuszewski et al 2021	Cognitive—tactile Braille learning	Repeated measures correlation	GM: R1
McPhee et al 2021	Cognitive—cognitive training with or without *Bacopa monnieri* supplementation	Correlated change	GM: NODDI-NDI, NODDI-ODI
Alharshan et al 2025	Perceptual—eye movement training	Correlated change	GM: DKI-MK, DKI-AK
Ziminski et al 2023	Perceptual—perceptual decision-making	Correlated change	GM: MT
Jünemann et al 2022	Music—piano training	Correlated change	WM: FBA-FD

All but one study reported correlated brain-behavior changes, whereas Matuszewski et al (2021) established “cross-sectional” brain–behavior correlations in the same sample at more than 1 time point. Reviewed studies that found correlations between more “conventional” MRI metrics such as DTI-FA or DTI-MD and learning metrics (e.g., Alwashmi et al 2025; Verhelst et al 2019) have not been considered in the table. AK, axial kurtosis; CHARMED, composite hindered and restricted model of diffusion; DDE, double-diffusion encoding; DKI, diffusion kurtosis imaging; FBA, fixel-based analysis; FD, fiber density; Fr, volume fraction of the restricted compartment; GM, gray matter; MK, mean kurtosis; MT, magnetization transfer; MWF, myelin water fraction; NDI, neurite density index; NODDI, neurite orientation dispersion and density imaging; ODI, orientation dispersion index; WM, white matter; μFA, microscopic fractional anisotropy.

It is also important to highlight that the reported associations should be interpreted cautiously, as they often emerge from modest sample sizes and heterogeneous behavioral measures, and in some cases, they occur in the absence of clear group-level microstructural effects. Such patterns may reflect substantial interindividual variability in training responses rather than reproducible mechanistic relationships (Thomas and Baker 2013; Hille et al 2024). Finally, most studies tacitly assume that brain and behavioral changes unfold concurrently. However, microstructural remodeling and behavioral improvement may occur on different time scales, and potential temporal lags have rarely been examined systematically (Rakowska et al 2025).

## Persistent Challenges and Future Directions

Recent advances in MRI hardware, preprocessing pipelines, and biophysical and statistical modeling have enabled substantial progress in human neuroplasticity research, as reflected in the studies reviewed herein. In the following, we discuss persistent methodological and inferential challenges, potential remedies, and key priorities for future work.

Since the critical review by Thomas and Baker (2013), the field has improved substantially with regard to the reproducibility and biological specificity of MRI-based imaging markers (Tardif et al 2016; Lerch et al 2017; Weiskopf et al 2021). However, several core challenges identified more than a decade ago remain largely unresolved. Most prominently, many studies continue to rely on small sample sizes and consequently limited statistical power (Szucs and Ioannidis 2020). Addressing this limitation might involve both strategic experimental design choices—such as within-subject control periods (Lehmann et al 2023; Ziminski et al 2023; Aye et al 2025; Aye et al 2026; Figure 4) or crossover designs with washout phases (Ueta et al 2022)—and systematic cohort scaling through larger samples or pooled analysis of raw data across studies or sites (Costafreda 2009; Weiskopf et al 2013). The adoption of harmonized MRI protocols and processing pipelines, along with broader adherence to community standards for data organization and sharing (e.g., Brain Imaging Data Structure), would further facilitate these efforts. Establishing more definitive evidence on microstructural plasticity will also require the consistent inclusion of appropriate control conditions and broader adoption of statistical modeling approaches that explicitly account for group × time interactions and mediation effects (Thomas and Baker 2013). These practices, however, remain insufficiently implemented.

**Figure 5.**
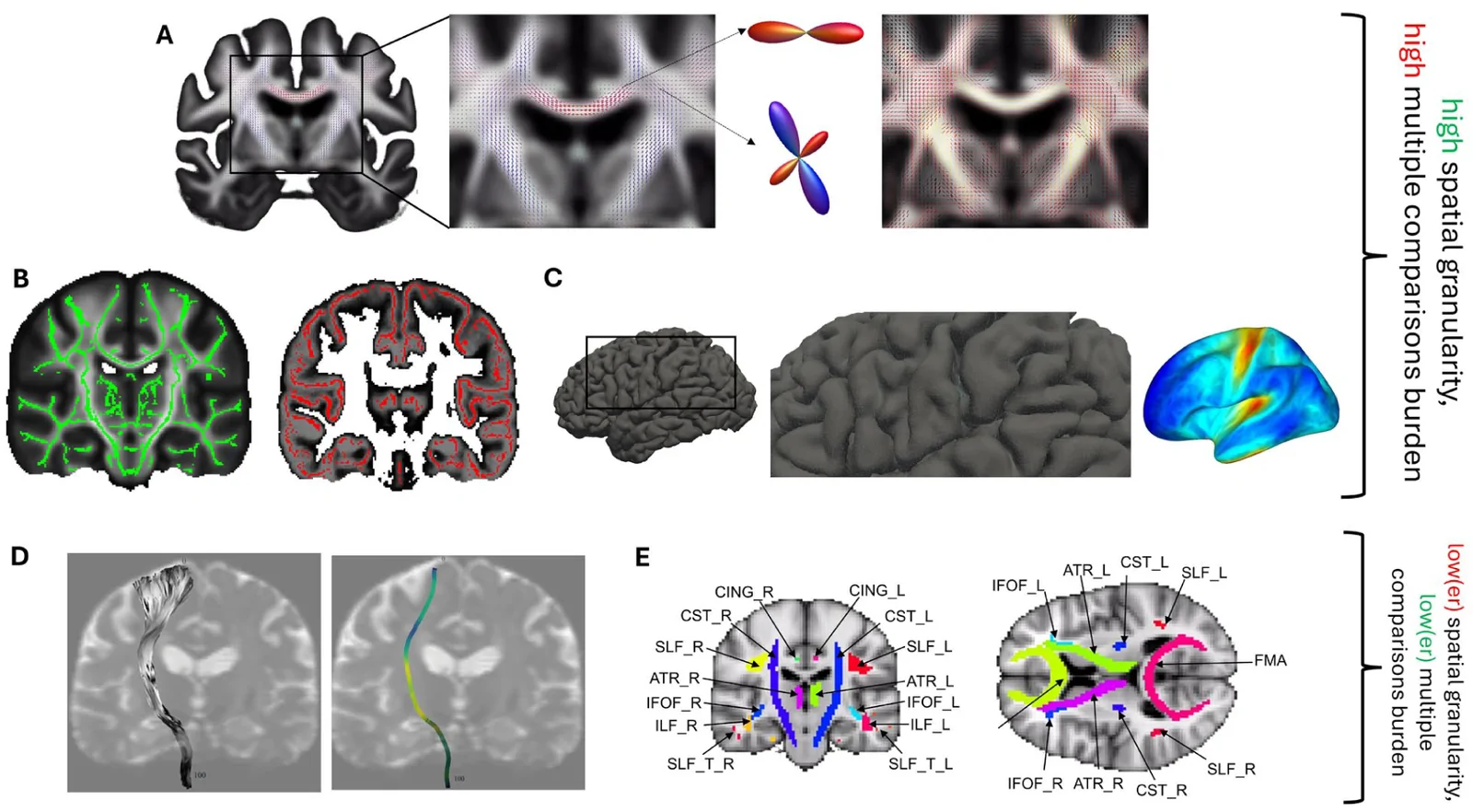
Analytical strategies for assessing microstructural plasticity and their trade-offs. Analytical approaches differ in spatial granularity and associated statistical burden. (A) In fixel-based analysis (FBA), fiber orientation distributions (FODs) are estimated per voxel. FOD shape differs between highly aligned tracts (e.g., corpus callosum) and regions with crossing fibers, such as the corona radiata. For FBA, FODs are segmented into distinct fiber populations (“fixels”). Color-coded fiber density (FD) shown on the right approximates intra-axonal volume for a fiber population, with yellow (red) representing high (low) values. Based on streamline tractography, FBA can assign statistical effects to specific fiber populations even in crossing-fiber regions. (B) Tract-based spatial statistics (TBSS) and gray matter–based spatial statistics (GBSS) as 2 examples of voxel-based quantification (VBQ) strategies. In these approaches, each subject’s local white or gray matter maxima are projected onto a common skeleton, enabling voxel-wise statistical comparisons across participants. (C) Surface-based analysis (SBA) performs statistical analysis on a reconstructed model of the cortical mantle represented as a triangular mesh. Microstructural metrics are projected from volumetric space onto this surface and analyzed at each mesh point (vertex). The color-coded surface shows the neurite density index (NDI) derived from neurite orientation dispersion and density imaging (from Taubert et al 2024). (D) Tractometry summarizes microstructural information from tractography-derived white matter pathways into a small set of averages (e.g., ~100 equidistant segments along a tract), on which statistical analysis is performed. E, Similarly, region-of-interest (ROI) analyses average signal across voxels within atlas-defined regions (e.g., the JHU white matter atlas). ROI analysis can be performed in either white or gray matter. Across approaches, increasing spatial granularity (A–C) entails mass-univariate testing across many spatial elements. More aggregated strategies (D, E) trade anatomic specificity for a reduced multiple-comparison burden and potentially greater statistical power.

A further longstanding methodological challenge concerns analytical strategy. It strongly influences anatomic specificity, statistical power, and ultimately reproducibility, yet the variability introduced by researcher degrees of freedom is only beginning to be systematically quantified (Botvinik-Nezer and Wager 2023). For example, approaches largely preserving spatial resolution—such as voxel-, surface-, or fixel-based analyses—incur substantial multiple-comparison penalties from mass-univariate testing, which are handled heterogeneously across studies. In contrast, region-of-interest or tract-segment averaging sacrifices spatial specificity in exchange for greater statistical power and robustness (Figure 5).

While advanced diffusion and relaxometry techniques are recognized for their improved biological specificity, their sensitivity to subtle microstructural changes, when compared to conventional MRI methods, remains a point of debate (Jelescu et al 2020). Some studies explicitly highlight enhanced sensitivity of advanced metrics (e.g., Tavor et al 2013; Li et al 2022; Economou et al 2023; Alharshan et al 2025), whereas others find conventional techniques, like DTI, outperforming biophysical models, such as NODDI, in detecting subtle hippocampal dendritic damage in pathological conditions (Crombe et al 2018). These mixed findings suggest that the 2 approaches are complementary rather than competing.

A promising route to increase sensitivity and reduce interpretational ambiguity is the use of multicontrast imaging (Zatorre et al 2012; Tardif et al 2016), coupled with analytic strategies capable of leveraging the complementary information provided by different MRI methods (Cercignani and Bouyagoub 2018). A few studies reviewed herein have applied this approach, employing techniques such as PCA (Metzler-Baddeley et al 2017; Casella et al 2020), multivariate analysis of variance (Matuszewski et al 2021), or multivariate extensions of linear mixed models (Aye et al 2026) to identify patterns of change or a nonparametric combination to test compound hypotheses (Rakowska et al 2025). These strategies have the potential to uncover structures in the data that single-metric analyses may overlook (Cercignani and Bouyagoub 2018), providing a more comprehensive and nuanced view of learning-related changes. Ultimately, such data fusion can help pinpoint the likely cellular mechanisms underlying MRI signal changes, refining hypotheses about the underlying biology (Tardif et al 2016; Cercignani and Bouyagoub 2018).

Future research will also benefit from linking microstructural plasticity to changes in structural connectivity and functional brain activation (Matuszewski et al 2021; Alwashmi et al 2025) and from cross-modal study designs. For example, transient disruption of M1 with TMS interfered with word learning and impaired learning-related microstructural changes in task-relevant regions (Vukovic et al 2021), demonstrating the causal involvement of the motor system in learning action-related language. Using combined MRI and magnetic resonance spectroscopy (MRS), Ziminski et al (2023) showed that subcortical myelin changes interact with visual-cortex GABAergic inhibition through altered thalamocortical connectivity, ultimately supporting improvements in perceptual decision-making and highlighting the potential of multimodal approaches to enrich our understanding of neuroplasticity.

In parallel, cross-species approaches and ultra-high-field MRI are continuing to offer valuable translational insights, helping bridge the gap between animal and human research. For instance, Faw et al (2021) identified activity-dependent oligodendroglial remodeling as a potential mechanism of rehabilitation-induced recovery in a combined human–animal study. Furthermore, the enhanced resolution of ultra-high-field MRI reduces partial-volume contamination and enables investigations at the laminar level. While a few learning studies, such as 1 DTI study on motor sequence learning (Tremblay et al 2021), have utilized 7T MRI, the use of microstructure-sensitive imaging at ultra-high-field strengths remains limited—but this is expected to grow in the future.

The temporal dynamics and long-term stability of microstructural reorganization during extended training are poorly understood, as most studies rely on pre–post MRI scans. Existing evidence suggests that both transient, nonlinear changes and steadily evolving, linear changes may occur (Huber et al 2021; Matuszewski et al 2021; Azzarito et al 2023; Emmenegger et al 2024), depending on the brain areas involved. However, these conclusions depend critically on the frequency of MRI measurements during the intervention and on the researchers’ assumptions regarding the trajectory of change. In addition, the concept of “neuroplasticity networks”—interconnected brain regions exhibiting similar change patterns, potentially with temporal delays between regions (Azzarito et al 2023; Emmenegger et al 2024; Aye et al 2026)—has only just begun to be explored. Future studies employing intensive longitudinal designs, with repeated measurements at closely spaced intervals (e.g., every few days), will be crucial for progress in this area.

As MRI methodology becomes more biologically informed, the field is shifting from merely describing brain changes to actively harnessing them. One area that has received comparatively little attention is interindividual variability in neuroplasticity. Learners differ substantially not only in their behavioral trajectories but also in the magnitude and temporal dynamics of microstructural plasticity (Hille et al 2024). For instance, negative correlations between putatively myelin-related changes in gray matter and behavioral changes suggest that individuals who face greater challenges during learning may experience more profound microstructural changes (Lakhani et al 2016; Ziminski et al 2023). While the moderators of such variability remain unclear, it is hypothesized that such differences interact with baseline brain status (Mizuguchi et al 2019; Huber et al 2021; Matuszewski et al 2021; Ueta et al 2022; Kyriazis et al 2025; Rakowska et al 2025), as well as sex (Kirby et al 2024), training volume/intensity (Brehmer et al 2014; Jünemann et al 2022), genetic background, and age (Brehmer et al 2014; Hille et al 2024). With few notable exceptions (Casella et al 2020; Liang et al 2021), it remains largely unexplored whether clinical populations exhibit neuroplastic responses that are comparable to, or distinct from, those observed in healthy control groups following similar interventions. Recently, the idea has emerged that certain neuroanatomical features that predict learning ability may be malleable through training, while others—such as cortical folding (Taubert et al 2024)—could function as more stable predispositions.

In addition to understanding the sources of interindividual variability, a key question for the future is whether this knowledge can be leveraged to tailor interventions to individual needs. Based on the exploration–selection–refinement framework of neuroplasticity (Lindenberger and Lövdén 2019), it can be hypothesized that plasticity is optimized when learners are challenged at an appropriate level, avoiding both under- and overload. Supporting this idea, Metzler-Baddeley et al (2017) demonstrated that individualized training outperformed nonadaptive training in promoting white matter reorganization and improving working memory performance. Complementary strategies aim to “prime” task-relevant circuitry before or during training, with early evidence suggesting that noninvasive brain stimulation (Antonenko et al 2023) and aerobic exercise (Lehmann et al 2022) can enhance learning-induced plasticity and improve behavioral outcomes.

Finally, it is important to acknowledge that most studies conducted to date have taken place in resource-rich settings—such as Europe, North America, East Asia, and Israel—and have primarily involved healthy, educated adults, with children or adolescents included only occasionally. As a result, these samples are unlikely to represent the global population in terms of ethnicity, age, education, or socioeconomic status. Future research should strive to include more diverse populations to evaluate the generalizability of findings on microstructural neuroplasticity (Falk et al 2013). This would help address the current overreliance on WEIRD (Western, Educated, Industrialized, Rich, Democratic) samples, which is largely driven by practical constraints such as the high cost and limited availability of MRI equipment.

## Conclusions

The flexibility of MRI to measure multiple tissue contrasts optimized for specific tissue features, combined with advanced modeling techniques, now permits increasingly detailed, in vivo characterization of the microstructural correlates of learning in humans. Longitudinal studies suggest that microstructural changes can occur within hours to days in response to learning, for example, in the hippocampus (Frankland and Bontempi 2005), with evidence of increased restricted diffusion—potentially reflecting more complex cellular processes—and local tissue volume. Restricted diffusion changes have also been observed after longer training periods, with additional involvement of myelin remodeling in task-relevant gray and white matter regions. However, the relationship between microstructural changes and behavioral improvements is complex and remains an area of ongoing investigation. As discussed, challenges such as temporal dynamics and variability in neuroplasticity responses need further exploration. It is hoped that continued advancements in MRI will bring us closer to actionable insights for real-world applications, such as enhanced diagnostic capabilities and the potential to individualize treatment plans based on neuroplastic principles.
